# Understanding and Addressing Variation in Health Care–Associated Infections After Durable Ventricular Assist Device Therapy: Protocol for a Mixed Methods Study

**DOI:** 10.2196/14701

**Published:** 2020-01-07

**Authors:** P Paul Chandanabhumma, Michael D Fetters, Francis D Pagani, Preeti N Malani, John M Hollingsworth, Russell J Funk, Keith D Aaronson, Min Zhang, Robert L Kormos, Carol E Chenoweth, Supriya Shore, Tessa M F Watt, Lourdes Cabrera, Donald S Likosky

**Affiliations:** 1 Mixed Methods Program Department of Family Medicine University of Michigan Ann Arbor, MI United States; 2 Department of Cardiac Surgery University of Michigan Ann Arbor, MI United States; 3 Division of Infectious Diseases Department of Internal Medicine University of Michigan Ann Arbor, MI United States; 4 Department of Urology University of Michigan Ann Arbor, MI United States; 5 Department of Strategic Management and Entrepreneurship Carlson School of Management University of Minnesota Minneapolis, MN United States; 6 Division of Cardiovascular Medicine Department of Internal Medicine University of Michigan Ann Arbor, MI United States; 7 Department of Biostatistics School of Public Health University of Michigan Ann Arbor, MI United States; 8 Department of Cardiothoracic Surgery University of Pittsburgh Medical Center Pittsburgh, PA United States

**Keywords:** heart failure, ventricular assist device, infection, cardiac surgical procedures, mixed methods

## Abstract

**Background:**

Durable ventricular assist device (VAD) therapy is reserved for patients with advanced heart failure who have a poor estimated 1-year survival. However, despite highly protocolized management processes, patients are at a unique risk for developing a health care–associated infection (HAI). Few studies have examined optimal strategies for HAI prevention after durable VAD implantation, despite variability in rates across centers and their impact on short- and long-term outcomes.

**Objective:**

The objective of this study is to develop recommendations for preventing the most significant HAIs after durable VAD implantation. The study has 3 specific aims: (1) identify determinants of center-level variability in HAI rates, (2) develop comprehensive understanding of barriers and facilitators for achieving low center-level HAI rates, and (3) develop and disseminate a best practices toolkit for preventing HAIs that accommodates various center contexts.

**Methods:**

This is a sequential mixed methods study starting with a cross-sectional assessment of current practices. To address aim 1, we will conduct (1) a systematic review of HAI prevention studies and (2) in-depth quantitative analyses using administrative claims, in-depth clinical data, and organizational surveys of VAD centers. For aim 2, we will apply a mixed methods patient tracer assessment framework to conduct semistructured interviews, field observations, and document analysis informed by findings from aim 1 at 5 high-performing (ie, low HAIs) and 5 low-performing (ie, high HAI) centers, which will be examined using a mixed methods case series analysis. For aim 3, we will build upon the findings from the previous aims to develop and field test an HAI preventive toolkit, acquire stakeholder input at an annual cardiac surgical conference, disseminate the final version to VAD centers nationwide, and conduct follow-up surveys to assess the toolkit’s adoption.

**Results:**

The project was funded by the Agency for Healthcare Research and Quality in 2018 and enrollment for the overall project is ongoing. Data analysis is currently under way and the first results are expected to be submitted for publication in 2019.

**Conclusions:**

This mixed methods study seeks to quantitatively assess the determinants of HAIs across clinical centers and qualitatively identify the context-specific facilitators and barriers for attaining low HAI rates. The mixed data findings will be used to develop and disseminate a stakeholder-acceptable toolkit of evidence-based HAI prevention recommendations that will accommodate the specific contexts and needs of VAD centers.

**International Registered Report Identifier (IRRID):**

PRR1-10.2196/14701

## Introduction

### Use of Durable Ventricular Assist Device to Treat Advanced Heart Failure

Heart failure affects nearly 5.7 million Americans and is a contributing cause of 1 in 9 deaths [1]. It is estimated that the prevalence of heart failure will increase by 46% from 2012 to 2030 [2]. The condition is the second costliest in terms of Medicare expenditures (approximately US $30.7 billion annually) [1]. Durable ventricular assist device (VAD) therapy is reserved for patients with advanced heart failure who have a poor estimated 1-year survival [3,4]. Technological advances using newer magnetically levitated centrifugal continuous-flow VADs have improved patient survival and decreased adverse event rates compared with older continuous-flow axial technology [5,6]. Currently, the estimated VAD survival is approximately 50% at 4 years, accompanied by significant improvements in functional status and quality of life [7,8].

### Risk of Device-Related Health Care–Associated Infection

Patients with VADs are at a heightened risk for device-related and nondevice-related health care–associated infection (HAI) despite a highly protocolized perioperative and postoperative course. The nature of durable VAD therapy requires an uninterrupted external power source connected to the patient through a driveline (ie, a percutaneous lead to provide power and control to the implantable pump), which serves as a potential source for the development of HAIs given its connectivity between the patient and the external environment [9]. In addition, VAD therapy is frequently associated with other infections that are nondevice-related, including pneumonia and surgical site or bloodstream infections [10,11]. VAD patients are at a unique risk for HAIs given (1) the burden of multiple preoperative comorbid conditions; (2) the invasive nature of VAD implantation; (3) hemodynamic instability that often occurs at the time of device implantation; (4) a common feature of all devices, a percutaneous lead; (5) extended intensive care unit stays; and (6) concurrent need for invasive monitoring.

The majority of HAIs occur within 90 days after a VAD implantation, with a decreased but ongoing risk beyond the 90-day period [12-16]. More than 3000 patients undergo VAD implantation in the United States annually, with nearly 6 out of every 10 patients developing a HAI following the procedure [16-22]. Development of an HAI is associated with a 6-fold risk of 1-year mortality and incurs additional treatment expenditures (US $264,000-US $869,000 per patient) [23,24]. Notably, infectious complications secondary to VAD implantation are the second leading cause of death for this population (16% of all deaths) [25].

### Variation in Health Care–Associated Infection and Preventive Strategies

Large variation exists in both the rate of HAIs and, more notably, the adoption of preventive strategies (eg, checklists, effective teamwork, and unit and center leadership) across clinical centers [26,27]. A 2017 study noted significant variation in the choice and duration of antimicrobial prophylaxis across 20 VAD centers (ie, one that implants a VAD or treats a patient within 90 days of implantation of a VAD) [26]. Although researchers have reported the benefit of preventive strategies, adoption by the clinical community varies [28-33]. Researchers have noted improved outcomes among cardiac surgery patients being cared at health systems with stronger provider teamwork (as captured in the configuration of social networks among providers), suggesting that a high level of provider teamwork may promote the prevention of HAIs [33,34]. Moreover, a 2018 study reported reductions in HAIs with the adoption of evidence-based practices within the setting of coronary artery bypass grafting procedures [35]. However, further investigation is required to understand the optimal HAI prevention strategies that have been adopted and how high-performing centers (ie, those with lower HAI rates) enhance the implementation of these strategies relative to low-performing centers.

### Conceptual Framework Guiding the Study

**Figure 1:**
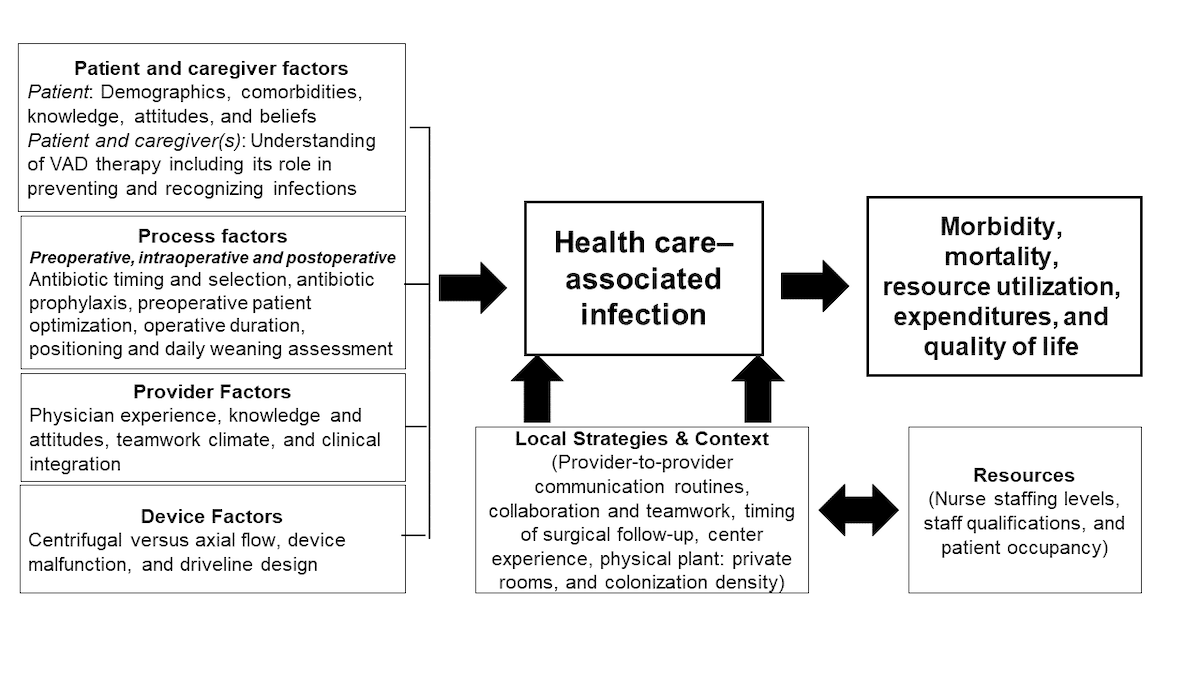
Conceptual model for health care–associated infection development and impact. VAD: ventricular assist device.

Figure 1 shows the conceptual framework illustrating the determinants of HAIs and informing the areas of investigation in this study. We hypothesized that patient and caregiver, process, provider, and device-related risk factors as well as a center’s local strategies, context, and resources are associated with the development of HAIs. Moreover, we hypothesized that, after accounting for perioperative risk factors, a center’s local strategies, context, and resources are associated with HAIs. Centers with lower HAI rates may leverage resources (eg, infection prevention staff) to reduce barriers to and increase facilitators of infection prevention strategies (eg, antimicrobial prophylaxis regimen) to prevent HAIs. Furthermore, we envision that a modular, action-oriented HAI toolkit, taking into account a center’s local strategies, context, and resources, could guide clinical teams (via evidence-based recommendations and educational tools) in preventing HAIs.

### Rationale for the Study

Few studies have focused on understanding both the optimal strategies for HAI prevention after VAD implantation as well as the approaches for enhancing the local adoption of these practices. Relying on administrative claims or clinical data is insufficient for identifying novel HAI practices, let alone understanding the characteristics of center- and unit-level strategies and contexts contributing to HAIs. To address this research gap, this mixed methods study seeks to identify recommendations for preventing the most clinically significant HAIs after VAD implantation. First, we plan to characterize key determinants of HAIs using quantitative approaches. Subsequently, we plan to identify context-specific promoters and barriers to preventing HAIs across low- and high-performing centers using qualitative approaches. More importantly, a multidisciplinary study team will use both findings to develop an action-oriented modular toolkit that provides evidenced HAI prevention recommendations, which accommodates the specific needs of individual centers. This project will contribute to research knowledge and interventions aimed at preventing HAIs by encouraging field-wide adoption of evidence-based practices.

## Methods

### Overall Study Design

**Figure 2:**
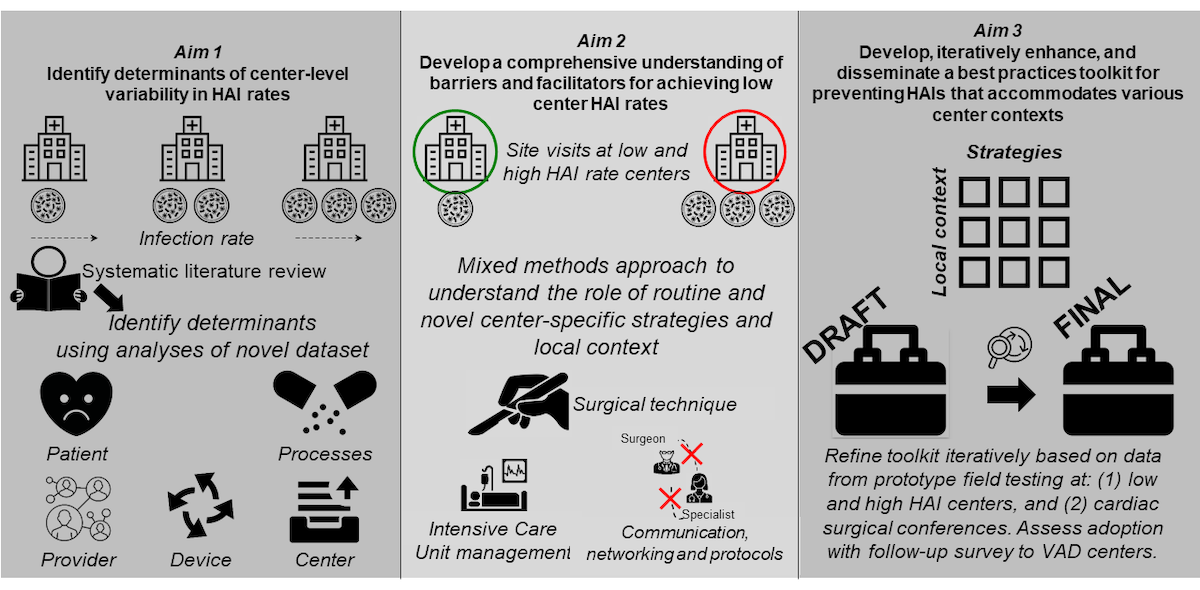
Overall study design. HAI: health care–associated infection; INTERMACS: Interagency Registry for Mechanically Assisted Circulatory Support.

As shown in Figure 2, we will use an explanatory sequential mixed methods design to address 3 aims: (1) identify determinants of center-level variability in HAI rates; (2) develop comprehensive understanding of barriers and facilitators for achieving low center-level HAI rates; and (3) develop, iteratively enhance, and disseminate a best practices toolkit for preventing HAIs that accommodates various center contexts. In aim 1, we will (1) conduct a systematic review of existing HAI prevention studies to compile a list of current prevention strategies and (2) conduct in-depth analyses using a unique merged dataset of all Food and Drug Administration (FDA)–approved VADs implanted in Medicare beneficiaries as well as an organizational survey administered to VAD centers. Our systematic literature review and analysis of provider social networks (including physician and nonphysician providers) will inform areas of investigation and identify the sample of high-performing and low-performing centers for aim 2. In aim 2, we will develop a comprehensive understanding of local facility and context-sensitive approaches contributing to variability in HAI rates through site visits at a sample of centers to reveal routine and novel HAI determinants and identify feasible solutions for ensuring local adherence to HAI prevention practices. In aim 3, we will (1) field test a prototype toolkit, (2) present an iteratively enhanced version to key VAD clinical stakeholders (eg, surgeons, clinical coordinators, nurses, and cardiologists) attending an annual conference in the field of cardiac surgery to optimize the toolkit’s usability and acceptability before national dissemination, and (3) conduct a follow-up survey to assess the adoption of the proposed toolkit. The human subjects’ applications for secondary use of the Interagency Registry for Mechanically Assisted Circulatory Support (INTERMACS) and Medicare data (HUM00155687) and for administration of center surveys (HUM00157335) have been approved by the institutional review board of the University of Michigan.

### Aim 1: Identify Determinants of Center-Level Variability in Health Care–Associated Infection Rates

To address this aim, we will first conduct a systematic review of interventional HAI prevention studies. Second, we will supplement the clinical dataset with (1) data from an electronically distributed survey of VAD centers to identify HAI prevention strategies not already tracked through INTERMACS and (2) center-specific measures of provider social network configurations using a merged INTERMACS-Medicare dataset [7,36]. Third, we will calculate center-specific 90-day HAI rates. Fourth, we will model variability in and identify determinants of centers’ HAI rates using this enhanced clinical dataset. We hypothesize that (after accounting for perioperative risk factors through INTERMACS) process, provider, device, and center- and unit-specific risk factors will be significant determinants of HAI rates. Findings will inform the sampling plan and focus on aim 2 activities.

#### Datasets Used for Aim 1

##### Center for Medicare and Medicaid Services Files

Given that Medicare is the largest US health care payer, Center for Medicare and Medicaid Services (CMS) is the sole national data source of exhaustive claims for noninstitutional and institutional providers. The CMS files contain data about (1) beneficiary (eg, age, diagnoses, and type of benefits); (2) institutional admissions; (3) provider services; (4) outpatient, hospice, home health agency, and skilled nursing facility services; and (5) prescription drugs (eg, heart failure medications). Each file contains data about the date and location of services and payments. We will characterize the social networks of providers (eg, nurse practitioners, physician assistants, and physicians) as a measure of provider teamwork. Following precedent from prior research on physician social networks, we will restrict these networks to specific provider specialties (eg, anesthesiology and surgery) that are most likely to be directly involved with the care of VAD patients [33,34,37-41]. Determination of relevant providers will be made through consultation with clinicians and through descriptive assessments of claims data.

##### American Hospital Association Survey

Data from this annual survey, to be merged with CMS files, contain center-specific structure and organizational measures (eg, size, urban or rural location, teaching status, affiliation with networks, training programs, ownership, staffing levels, and types of surgical services provided).

##### Interagency Registry for Mechanically Assisted Circulatory Support

INTERMACS is a multicenter, Joint Commission–recognized, nationally audited database of FDA-approved VADs. INTERMACS contains extensive data regarding (1) preimplant details (eg, age and comorbid diseases); (2) operative details (eg, device type and operative duration); (3) patient status until death, cardiac transplantation, or device removal; (4) adverse events (eg, device malfunctions); and (5) functional status, neurocognition, and health-related quality of life. INTERMACS monitors device-related (driveline, exit cannula, pump pocket, and pump interior) and nondevice-related (positive blood cultures, central line associated sepsis, pulmonary, urinary tract, mediastinum, peripheral wound, gastrointestinal) HAIs.

##### Center Survey

The survey, informed by a systematic review of the literature and prior work, will identify potential HAI determinants that are not available through existing data sources [42,43]. Independent of our network analysis, the survey will be designed to address determinants spanning the patient’s full clinical trajectory (preoperative, implantation, postoperative, rehabilitation, and discharge). Surveys will be sent to the center’s designated VAD coordinator, implanting surgeons, and heart failure cardiologists involved in the center’s VAD program. The use of Web-based survey software (ie, Qualtrics) will allow for the creation, distribution, storage, and analysis of survey instruments, with advanced features (eg, randomization of question ordering).

Preliminary survey drafts will be developed based on a conceptual framework and by our investigative team of clinical and research experts, with input from survey and decision science experts. We will test the draft survey instrument with key stakeholders at selected centers with low and high HAI rates to ensure readability, face validity, and completeness of response options. Interviews will be conducted by Web-based teleconference in real time as the draft survey is completed to solicit qualitative feedback on survey content. The survey will be revised based on this feedback.

We will use evidence-based strategies to maximize survey response rate and minimize nonresponse bias, especially as a function of a center’s HAI rate. For example, the email invitation will be personalized, and the survey length will be limited. In addition, nonrespondents (VAD coordinator, surgeon, and cardiologist) will be contacted by the study team as necessary. Our team will provide respondents with a small gift card after completing the survey.

#### Sample Size

We project analyzing data among 9339 VAD patients receiving durable VAD from 2008 to 2017. We will administer surveys to potential respondents (VAD coordinator, surgeon, and cardiologist) from an estimated 153 VAD centers.

#### Analytic Approach

First, we will conduct a systematic review of the published literature to identify established HAI preventive practices [44,45]. Second, we will use survey responses to identify provider and institutional practices that contribute to center variability in HAI rates. Third, we will use our linked Medicare-INTERMACS datasets to identify preoperative, intraoperative, and postoperative factors that contribute to center variability in HAI rates.

##### Primary Endpoint

Our primary endpoint is any HAI 90 days after device implant. We will account for HAI competing risks (eg, death and device removal) and other censoring (follow-up less than 90 days) using time-to-event analysis. Furthermore, we will use 2 approaches for modeling HAI risk. First, we will use the Cox proportional hazards model. We will adjust for surgery year and season and investigate interactions by key biological variables (eg, patient age, sex, and race). We will account for patient demographics (eg, age and sex), disease characteristics (eg, pulmonary disease), and surgical history (eg, prior cardiac surgery) and study the effect of intraoperative care (eg, cardiopulmonary bypass duration) and postoperative care (eg, duration of intubation). We will use the Lasso variable selection method to assess variables for model inclusion [46]. We will handle the multilevel structure (eg, patients are nested within centers) using the robust sandwich variance–based inference or frailty model approach. On the basis of the final fitted model, effects of risk factors will be quantified by hazards ratios and tested by score tests. Second, we will use random survival forest, an extension of the random forest method to right-censored time-to-event data, to model the effects of risk factors nonparametrically [47-49]. The final model will be an ensemble average of fitted trees from bootstrap samples.

##### Exploratory Analyses

We will also conduct exploratory analyses for device-related and nondevice-related HAIs and their main components (to address heterogeneity of risk factors across HAI subtypes). We will quantify the importance of a group of factors in explaining center variability in HAI by fitting models with and without such factors and comparing how well the expected numbers from different models track with the observed numbers. Primary analyses will include all centers, and for the purpose of sensitivity analysis, we will also exclude low-volume centers. We will explore different center volume thresholds, trying to strike a balance between 2 considerations: stable estimate of HAIs rate (quantified by standard error) and number of centers remaining for analysis. We will use simple mean imputation, median imputation, or multiple imputation to account for anticipated missing covariates depending on their pattern and mechanism [50]. If the missing data itself are thought to be related to outcomes, then missing indicators will be used and modeled as independent variables as part of sensitivity analysis.

##### Teamwork and Communication as Quality Measurement

Provider social networks represent a measure of teamwork and communication that are associated with the quality of care [34]. In prior validation work, shared patients between providers, identified using unique provider identifiers found in claims data, have been found to correspond closely to surveyed provider network patterns [33,51]. We will characterize mapped VAD networks using a measure known as assortativity, which can capture the degree to which providers from different specialties and clinical disciplines are interconnected and, thus, better equipped for collaboration and teamwork [52]. We will incorporate this measure as a covariate in our statistical models [34]. Recent network research has drawn attention to several challenges in characterizing assortativity in social networks. First, global (ie, network level) measures of assortativity may mask considerable local (ie, node level) variation in cross-group (eg, specialty) interaction [53]. Second, common network data practices (eg, unipartite projection of a bipartite network) may lead to bias in values of standard assortativity measures [54]. To overcome these challenges, we plan to use state-of-the-art techniques for evaluating assortativity in social networks, including multiscale measures of assortativity (which allow for measuring assortativity at the node level) and comparison of assortativity on our observed networks with null (ie, random network) models.

Claims data do not map perfectly to true care patterns; therefore, our network measures may include false negatives and false positives. To assess for these biases, we will run several sensitivity analyses, motivated by prior work on missing data and thresholding techniques in the study of social networks [55-57]. Using Monte Carlo simulations, we will randomly drop increasingly larger fractions of providers and relationships in our observed networks, recompute our network measures, and then rerun our statistical models to assess bias attributed to false negatives. In additional simulations, we will also gradually add larger fractions of random providers and relationships to our observed networks to assess sensitivity to false positives.

The approaches discussed above consider the time-to-event endpoint (ie, time to first HAI), which counts multiple HAIs from a single patient only once. Alternatively, we will consider the recurrent event endpoint, where multiple HAIs from a single patient are counted multiple times. We will use a counting process, Anderson-Gill model, to rerun the analysis using a recurrent event endpoint to help distinguish the 2 outcome scenarios [58].

### Aim 2: Develop a Comprehensive Understanding of Barriers and Facilitators for Achieving Low Center Health Care–Associated Infection Rates

A mixed methods patient tracer assessment framework will be applied (adapted from the Joint Commission’s tracer methodology) in aim 2 to examine center-level resources and local barriers and facilitators for achieving low HAI rates [59]. We will conduct semistructured interviews, field observations, and document analysis at 5 high-performing (ie, low HAI) and 5 low-performing (ie, high HAI) centers, which we will examine as a mixed methods case series to address this aim.

#### Sampling

A priori, we will use HAI rates derived from aim 1 to intentionally sample 5 high-performing centers with low HAI rates and 5 low-performing centers with high HAI rates for site visits. However, the sampling process will be further refined based on other criteria pending the nature of findings emerging from aim 1 and center-specific practices (eg, measures of provider teamwork).

#### Data Collection

We will conduct 2-day site visits at 5 high-performing and 5 low-performing centers. The mixed methods patient tracer assessment procedure focuses on the patient’s trajectory from the index hospitalization to 90 days. Before site visits, we will conduct advanced analytics to examine performance features, measures, and other quantitative data that will inform qualitative data collection. During the center visits, the site-visiting team will systematically trace the patient’s movement through the health system and investigate HAI risk factors or preventive strategies at each *stop* of the patient’s trajectory (ie, transitions in care or changes in the patient’s physical location). At each site, we will (1) conduct in-depth, semistructured interviews with relevant provider and nonprovider stakeholders and perform field observations of the environment and staff behaviors in the clinical unit and relevant operating room; (2) collect, discuss, and analyze relevant protocol documents; (3) learn local strategies used to enhance HAI prevention; and (4) gather toolkit design elements that would enhance its receptivity and local adoption. These findings will provide a robust understanding of the organizational resources and local facility barriers and facilitators for HAI prevention.

##### Semistructured Interviews

The site-visiting team will follow a patient’s trajectory and conduct semistructured interviews with stakeholders impacting VAD patient care, including administrators (eg, chief medical or nursing officer, quality or safety officer, physician service chief, clinical unit manager, and hospital epidemiologist), physicians (eg, attending surgeon, intensivist, hospitalist, and medical consultants—pulmonologist and cardiologist), advanced practice providers (eg, nurse practitioner and physician assistant), and nurses (eg, ward and intensive care unit). Informed consent will be obtained from informants before starting each interview. Each interview, which will be conducted in private offices or conference rooms, will last for 40 min to 60 min. Stakeholder interviews will focus on answering questions from the advanced analytics, understanding perceptions of the center’s resources and local strategies for HAI prevention, and eliciting key features that would enhance local toolkit adoption. Interviews will continue until reaching informational redundancy or saturation (ie, no new information is being identified) at each center. Following each interview, the study team will provide a gift certificate to each interviewee to acknowledge his or her contribution.

##### Observations and Field Notes

For each site visit, the site-visiting team will conduct direct observations of the clinical work environment and behavior within each *stop* of the patient trajectory. Quantitative observations regarding HAI determinants will be tracked using a structured data form. For example, we will track discrete process of care (eg, antibiotic prophylaxis regimens and preoperative optimization), provider practices (eg, location of driveline exit site), local context and strategies (eg, sink in every patient room and private vs shared patient rooms), and organizational resources (eg, nursing staffing levels). Qualitative observations will focus on examining (1) *context*—circumstances informing data collection (eg, recent line infection mortality); (2) *content*—factual data, locations visited, key process stakeholders, and ward layouts; and (3) *concepts*—hunches or theories about the environment that may help explain a center’s HAI rate (eg, deviations from written protocols) [60].

##### Documents

During each site visit, we will collect relevant protocol documents and interview participants at the relevant transition point about the use of the documents, variations, or other volunteered information. Other relevant documents, patient education documents, postsurgical order sets, and variations by surgeons will be collected.

#### Analytical Approach

##### Qualitative Analysis

Preliminary analysis will begin with a research team debrief at the end of each day, which will reflect team members’ field notes, impressions, and observations. These recorded conversations will provide an additional source of data for defining facility- and unit-level contextual characteristics. Debriefing sessions during day 1 will also identify areas of focus and inquiry for day 2. On day 2, we will explore questions raised on day 1 to develop an expanded understanding. At the end of day 2, the most current version of the toolkit will be presented to the sites for feedback.

##### Developing Case Studies

For each site, we will develop a case study following a structured outline that will have a sufficiently flexible format to accommodate site variation [61]. As the study summary for each site is completed, we will compare the findings with the other sites for the case series analysis. For each site case study, the summary will begin following each visit, when team members will review all qualitative and quantitative sources of data from each site. Team members will then begin the process of coding all the qualitative data including deidentified transcripts using qualitative analysis software (MAXQDA). Team members will convene regularly to compare independent coding of the qualitative data and revise the codebook iteratively until consensus is reached on codes, themes, categories, and coding criteria. This group consensus approach facilitates, enriches, and increases the rigor of data interpretation [62]. The study team will (1) develop findings by meeting regularly to review code summaries and memos created during prior meetings and (2) discuss and interpret the data across low- and high-performing centers. The analysis will occur by site, and findings or new questions will be iteratively explored after completion of each site visit.

#### Integration of Quantitative and Qualitative Analyses

All quantitative (aim 1 findings about the specific sites) and qualitative data (aim 2 findings) will be integrated into the case study for each site. Related findings from both data sources will be matched to provide a comprehensive understanding of each center’s HAI prevention strategies. Multiple case series analyses will be performed. As appropriate for related findings, joint display analysis (ie, the process of iteratively creating, interpreting, and restructuring tables and figures that integrate the quantitative and qualitative findings) will be used to illuminate similarities and differences in HAI prevention strategies across centers and draw overall conclusions [63].

#### Member Checking

Member checking is the process of providing qualitative and mixed methods findings back to study participants to elucidate their input on the overall interpretation [64]. The case study summary for each site will be distributed back to participants who indicated an interest in reviewing the findings. These participants will be asked to indicate their overall agreement with the study findings and to make any corrections or clarifications they deem necessary. Any requisite correction or clarification will be incorporated into the final case study reports used in the case series analysis.

### Aim 3: Develop, Iteratively Enhance, and Disseminate a Best Practices Toolkit for Preventing Health Care–Associated Infections That Accommodates Various Center Contexts

The study team will create a HAI preventive toolkit of evidence-based recommendations that may be customized to the context of each center. The development of the toolkit will be informed by the findings from aims 1 and 2. We will field test our prototype toolkit at the same 10 centers that participated in aim 2. This process will identify approaches for optimizing adoption (ie, fit and suitability for everyday use) as well as inform local context needs and any necessary modifications (eg, content, design, and options). The team will host a dedicated 90-min session at an annual cardiac surgical conference (eg, the International Society for Heart and Lung Transplantation) to elicit further user feedback about the toolkit’s acceptability. We will use the feedback to develop a final toolkit, which will be distributed to US VAD centers. Finally, a follow-up survey will be electronically distributed to US VAD centers to assess national adoption rates of the toolkit.

#### Translation of Findings Into a Draft Toolkit

**Figure 3:**
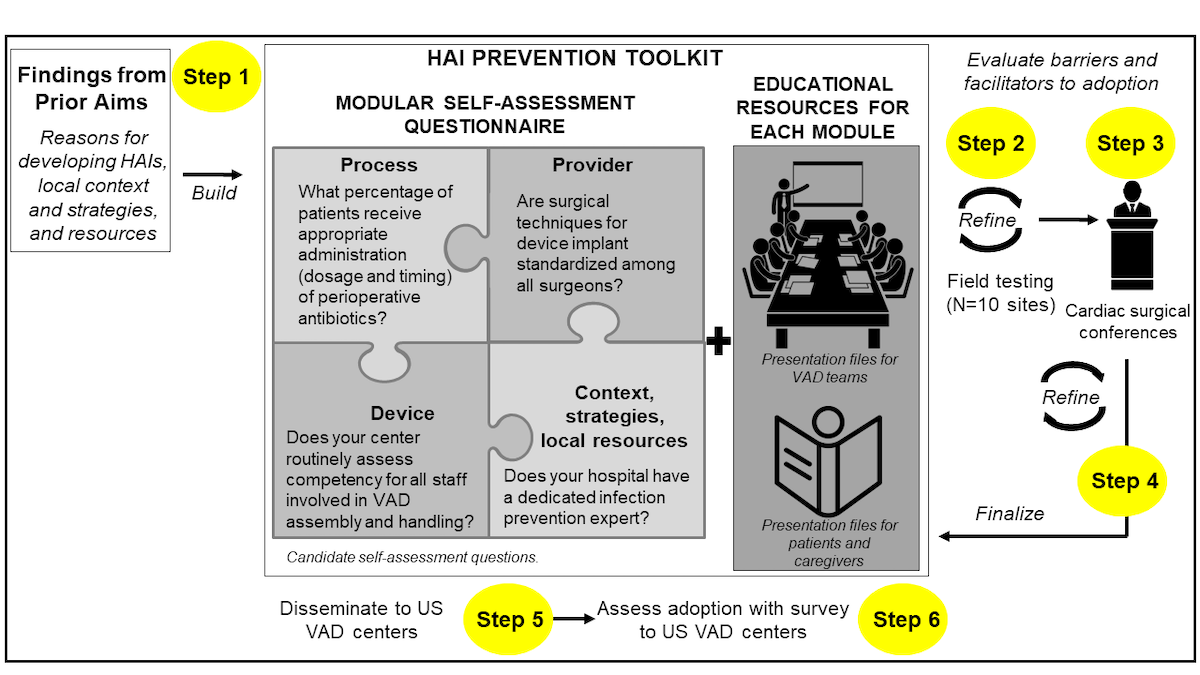
Development of the health care–associated infection preventive toolkit. HAI: health care–associated infection; INTERMACS: Interagency Registry for Mechanically Assisted Circulatory Support; VAD: ventricular assist device.

We will develop an initial version of a printed HAI prevention toolkit of evidence-based recommendations based on the Agency for Healthcare Research and Quality (AHRQ) guidelines [65]. In addition, we anticipate using the RE-AIM framework when considering the components of our toolkit as they relate to the likelihood of reach, efficacy, adoption, implementation, and maintenance [66]. The steps involved in the development of the toolkit are illustrated in Figure 3. The content of the toolkit will be informed by the findings from aim 1 (determinants of center’s HAI rates) and aim 2 (center’s strategies, context, and local resources). The toolkit will be designed to provide local customization to optimize adoption by individual centers. The toolkit will include a self-assessment questionnaire and corresponding educational resources. The self-assessment questionnaire will be designed to locally tailor evidence-based recommendations. The set of educational resources derived from stakeholder interviews will also address local, context-specific needs. We anticipate that the user-friendly toolkit will provide educational resources (eg, impact, prevention, pathophysiology, and epidemiology), prevention strategies (eg, concise summary of evidence-based recommendations with supporting literature).

#### Field Testing of the Toolkit

The prototype toolkit will be field tested at the same 10 centers that participated in aim 2. Study team members will conduct audio-recorded interviews of VAD clinical stakeholders to elicit user feedback regarding the toolkit’s usability, acceptability, and likelihood of significant effectiveness for preventing HAIs using a *think out loud* approach [67]. Although primarily intended to identify approaches for optimizing adoption (ie, fit and suitability for everyday use), the interviews will inform local context needs and any necessary toolkit modifications (eg, content, design, and options). Study team members will analyze field notes and deidentified transcripts. Iterative versions of the toolkit will be developed and presented at subsequent centers, thus enhancing local stakeholder acceptability and usability. After iterative development across the 10 centers, a fully revised and enhanced version will be sent back to the centers for final review and feedback. A further refined version will be prepared for feedback and presentation at an annual cardiac surgical conference (eg, the International Society for Heart and Lung Transplantation).

#### Broad Ventricular Assist Device Stakeholder Input on the Toolkit

A moderated expert panel will be convened at an annual cardiac surgical conference (eg, the International Society for Heart and Lung Transplantation) to gather stakeholder input on the refined toolkit. During this session, we will share the rationale and intended use of the toolkit and highlight the toolkit’s usability, acceptability, and likelihood of significant effectiveness. Attendees will use an audience response system to provide Likert-scaled responses to each question and will be invited to offer additional qualitative feedback concerning the reasons underlying their quantitative survey responses. Panelists (representing cardiac surgery, heart failure cardiology, infectious disease, and epidemiology) will be invited to respond to attendees’ remarks. The entire session will be double audiotaped (with deidentified transcripts created thereafter). Analysis of the audience response system data and the themes emerging from the discussion for each item will be integrated to draw overall conclusions and inform final revisions of the toolkit. Thereafter, we will distribute the final version to US VAD centers.

#### Assessment of Toolkit Adoption

A follow-up survey intended to assess toolkit adoption rates among US VAD centers will be developed and electronically distributed using the same approach as the center survey described in aim 1. The survey will be pretested to assess face and content validity, comprehensibility, time to completion, and ambiguity. The survey is anticipated to solicit information using a mixture of multiple-choice responses (eg, awareness of the toolkit, roles of those involved with local adoption, identified surgeon champion, types of resource support provided to the adoption team, and frequency of team meetings) and open-ended responses (eg, method used to implement the toolkit, perceived barriers and facilitators for implementation, ongoing quality improvement initiatives to enhance local adoption, and perceived effectiveness of the toolkit).

## Results

The project was funded by the AHRQ in 2018 and enrollment for the overall project is ongoing. We are conducting a systematic review of interventional HAI prevention studies and developing the survey concerning HAI determinants across US VAD centers. We anticipate that survey data collection will begin in November 2019. Findings from aims 1 and 2 will be used to develop a toolkit of evidence-based HAI prevention practices that may be adopted to the local contexts and across VAD centers. The first results are expected to be submitted for publication in 2019.

## Discussion

### Strengths

Although current emphasis is placed on surgical technical competence and checklists, further improvements in patient safety and outcomes may only be achieved with greater attention to optimizing the organization of clinical practice to reliably deliver safe and effective care. Our mixed methods study has several strengths. First, we will employ network analytic tools to assess whether provider teamwork is a determinant of variation in center-level HAI rates. This analytic method will enable us to account for differences in collaboration and communication across provider teams that would not be captured through traditional patient risk factors. Second, we will employ a novel patient tracer mixed methods assessment in which center-specific outcomes inform our qualitative investigation as we follow a hypothetical patient through each critical transition (or stop) of the patient’s care trajectory. Using this novel technique, we will identify potentially modifiable contexts, communication, and practices that could be missed if solely relying on quantitative approaches. Third, we will enhance our toolkit’s adoption by field testing a prototype during site visits to assess end-user usability and adoption, incorporating broad provider community input at an annual cardiac surgical conference (eg, the International Society for Heart and Lung Transplantation) before national dissemination and conducting follow-up surveys to assess the uptake of the toolkit.

### Limitations

Although unlikely, there are a few unanticipated challenges with this study. First, it is possible that we will not identify determinants of HAI across centers. There is a possibility that we will not find distinct HAI determinants across centers. However, given the documented center variability in anticoagulation practices and pump implant techniques, we anticipate ample variability related to HAI prevention practices [68]. Second, in the case of unobserved variability in HAI rates, we may have to adjust our qualitative sampling frame to include other relevant characteristics where variability is likely. However, we will use maximum variation sampling among the 5 low-performing and 5 high-performing centers to adjust the selection of sites to incorporate other selection criteria (eg, center volume and strength of provider network) [62]. Third, we may encounter unanticipated issues regarding the toolkit receptivity by US VAD centers. However, we will use the iterative stakeholder testing process to incorporate context-specific elements and ensure broad generalizability of the toolkit.

### Conclusions

This study seeks to elucidate determinants of HAI across clinical centers using quantitative approaches and identify context-specific facilitators and barriers for attaining low HAI rates using qualitative approaches. We will use these findings to develop and disseminate a stakeholder-acceptable toolkit of evidence-based HAI prevention recommendations that will accommodate the specific needs of VAD centers and address AHRQ patient safety guidelines [65]. The overall mixed methods approach may offer an investigative model for evaluating and improving clinical care, particularly in the area of complex surgical procedures.

## Abbreviations

AHRQ: Agency for Healthcare Research and Quality
CMS: Center for Medicare and Medicaid Services
FDA: Food and Drug Administration
HAI: health care–associated infection
INTERMACS: Interagency Registry for Mechanically Assisted Circulatory Support
NHLBI: National Heart, Lung, and Blood Institute
NIH: National Institutes of Health
VAD: ventricular assist device
