# Authorship Correction: Understanding and Addressing Variation in Health Care–Associated Infections After Durable Ventricular Assist Device Therapy: Protocol for a Mixed Methods Study

**DOI:** 10.2196/18324

**Published:** 2020-06-11

**Authors:** P Paul Chandanabhumma, Michael D Fetters, Francis D Pagani, Preeti N Malani, John M Hollingsworth, Russell J Funk, Keith D Aaronson, Min Zhang, Robert L Kormos, Carol E Chenoweth, Supriya Shore, Tessa M F Watt, Lourdes Cabrera, Donald S Likosky

**Affiliations:** 1 Mixed Methods Program Department of Family Medicine University of Michigan Ann Arbor, MI United States; 2 Department of Cardiac Surgery University of Michigan Ann Arbor, MI United States; 3 Division of Infectious Diseases Department of Internal Medicine University of Michigan Ann Arbor, MI United States; 4 Department of Urology University of Michigan Ann Arbor, MI United States; 5 Department of Strategic Management and Entrepreneurship Carlson School of Management University of Minnesota Minneapolis, MN United States; 6 Division of Cardiovascular Medicine Department of Internal Medicine University of Michigan Ann Arbor, MI United States; 7 Department of Biostatistics School of Public Health University of Michigan Ann Arbor, MI United States; 8 Department of Cardiothoracic Surgery University of Pittsburgh Medical Center Pittsburgh, PA United States

The authors of "Understanding and Addressing Variation in Health Care–Associated Infections After Durable Ventricular Assist Device Therapy: Protocol for a Mixed Methods Study" (JMIR Res Protoc 2020;9(1):e14701) noticed errors in the author list after publication.

Author Donald Likosky's name was missing the middle initial. Their name has been revised to "Donald S Likosky". Additionally, there were errors in some of the author degrees. Preeti N Malani's degrees have been revised from "MSCJ, MD" to "MSJ, MD", Tessa M F Watt's degrees have been revised from "MD" to "MSc, MD", and Keith D Aaronson's degrees have been revised from "MSc, MD" to "MS, MD".

The correction will appear in the online version of the paper on the JMIR website on June 11, 2020, together with the publication of this correction notice. Because this was made after submission to PubMed, PubMed Central, and other full-text repositories, the corrected article has also been resubmitted to those repositories.

